# Ampreloxetine Versus Droxidopa in Neurogenic Orthostatic Hypotension: A Comparative Review

**DOI:** 10.7759/cureus.38907

**Published:** 2023-05-11

**Authors:** Pranvera Hoxhaj, Shruti Shah, Veronica E Muyolema Arce, Wajiha Khan, Amirali Sadeghzadegan, Saumya Singh, Gaudy F Collado, Abhishek Goyal, Imran Khawaja, Deepti Botlaguduru, Waleed Razzaq, Zain U Abdin, Ishita Gupta

**Affiliations:** 1 Internal Medicine, Scher & Kerenyi MDS, New York, USA; 2 Internal Medicine, Byramjee Jeejeeboy (BJ) Medical College, Pune, IND; 3 Medicine, Universidad de Guayaquil, Guayaquil, ECU; 4 Oncology, Ziauddin University, Karachi, PAK; 5 Internal Medicine, Marmara University School of Medicine, Istanbul, TUR; 6 Internal Medicine, Gujarat Medical Education & Research Society (GMERS) Medical College and Hospital, Gujarat, IND; 7 Internal Medicine, Fleet Medical Unit, Philippine Fleet, Philippine Navy, Cavite City, PHL; 8 Internal Medicine, Kasturba Medical College, Manipal, Manipal, IND; 9 Internal Medicine, Ayub Medical Institute, Abbottabad, PAK; 10 Internal Medicine, MediCiti Institute of Medical Sciences, Mulugu, IND; 11 Internal Medicine, Services Hospital Lahore, Lahore, PAK; 12 Medicine, District Head Quarters Hospital, Faisalabad, PAK; 13 Medicine, Dr. Rajendra Prasad Government Medical College, Tanda, IND

**Keywords:** parkinson's disease, review, neurogenic orthostatic hypotension, droxidopa, ampreloxetine

## Abstract

Neurogenic orthostatic hypotension (nOH) is a disabling problem of autonomic dysfunction in patients with Parkinson’s disease, which is associated with poor quality of life and higher mortality rates. The purpose of this literature review was to explore and compare the efficacy and safety of droxidopa (an existing treatment) and ampreloxetine (a newer medication) in the treatment of nOH. We used a mixed-method literature review that addresses the epidemiology, pathophysiology, and pharmacological and non-pharmacological management of nOH in Parkinson’s disease in a general way, with a more exploratory approach to droxidopa- and ampreloxetine-controlled trial studies. We included a total of 10 studies of randomized controlled trials with eight studies focused on droxidopa^ ^and two studies focused on ampreloxetine. These two drugs were analyzed and compared based on the collected individual study results. Treatment of nOH in Parkinson’s disease patients with droxidopa or ampreloxetine showed clinically meaningful and statistically significant improvements relative to placebo on the components of the OHSA (Orthostatic Hypotension Symptom Assessment) composite score and OHDAS (Orthostatic Hypotension Daily Activity Scale composite scores) composite score. Droxidopa had an improved effect on daily activities, with an associated increase in standing systolic blood pressure (BP), but the long-term efficacy of droxidopa has not been documented. Standing systolic BP was maintained by ampreloxetine and worsened after the withdrawal phase. This highlights the importance of conducting further research which will help us to improve the therapeutic approach for patients with nOH and Parkinson’s disease.

## Introduction and background

Parkinson’s disease (PD) is a progressive neurodegenerative disease of the autonomic nervous system. This causes impairment and/or death of nerve cells in the basal ganglia, which causes the production of less dopamine and loss of nerve endings that produce norepinephrine [1]. Motor symptoms include slow movements, tremors, gait, and balance disturbances, while non-motor symptoms include orthostatic hypotension (OH), constipation, urinary disturbances, sleep disorders, and a spectrum of neuropsychiatric symptoms like dementia, hallucinations, etc. [2,3]. Lightheadedness, generalized weakness, dizziness, fading vision, loss of consciousness, and recurrent falls are some manifestations of OH [4]. The current definition of OH based on expert consensus is a fall of at least 20 mmHg in systolic blood pressure (BP) or 10 mmHg in diastolic BP within three minutes of standing or upright tilt [5,6]. It arises when there is a failure of the autonomic compensatory mechanism to maintain upright blood pressure along with reduced norepinephrine postganglionic release. This causes abnormal vasoconstriction while assuming an erect posture [7].

PD roughly has an impact on one in 1000 people in the community [8]. According to recently published studies, approximately 30%-50% of patients with PD suffer from OH [9-11]. The prevalence of OH increases with age and disease progression [12]. OH was the major cause of falls in 4.1% of PD patients in 2013 [13]. One study followed up 136 patients with PD over 20 years and found that 48% of the candidates had symptomatic OH, and 87% experienced falls, some of which could have been attributed to OH [14].

The goal of neurogenic OH management is to reduce symptoms, decrease the risk of falls and fall-related injuries, and enhance the quality of life by improving patients' abilities and confidence to perform activities independently [4,15,16]. These can be accomplished by correcting aggravating factors followed by consideration of non-pharmacologic and pharmacologic therapies [4,15].

Before beginning any pharmacological treatment, the patient’s medications should be carefully reviewed [4]. Neurogenic orthostatic hypotension (nOH) may be exacerbated by drugs that decrease intravascular volume (diuretics), induce vasodilatation (sildenafil and nitrates), or block norepinephrine release/activity at the neurovascular junction (α-blockers, centrally acting α2-agonists, and tricyclic antidepressants) [4,15,17]. Additionally, medications for Parkinson's disease, including levodopa, dopamine agonists, monoamine oxidase inhibitors, and amantadine, may also lower the BP [4,15-17]. Further, treatment of dehydration, infections, or anemia is also done to combat nOH [17].

Various lifestyle measures like standing up slowly from a supine position, using an elastic belt (abdominal compression), performing BP-raising maneuvers such as legs crossing, tensing the gluteal and abdominal muscles, drinking a bolus of water (approximately 500 mL in 5 min), and avoiding valsalva-like maneuvers during micturition or bowel movements can effectively prevent BP drops [15-17].

When medication adjustments and nonpharmacologic management fail to alleviate nOH symptoms, pharmacologic therapy is used [4,15-17]. Droxidopa is a norepinephrine prodrug that is converted into noradrenaline [4,16,17]. The US Food and Drug Administration (FDA) has approved droxidopa for the treatment of symptomatic nOH [4,15-17]. Extensive clinical experience demonstrates that droxidopa is a safe and well-tolerated drug that improves dizziness/lightheadedness and decreases falls caused by nOH through increased standing systolic blood pressure [4,15]. The typical dosing of droxidopa is 100-600 mg three times during the day with a 2.5-hour half-life [15,17].

Ampreloxetine is a selective norepinephrine reuptake inhibitor that increases norepinephrine bioavailability at the neurovascular junction. This newer drug has been found to have a longer plasma half-life and stable plasma levels over 24 h suggesting that it could be an effective treatment for nOH. Common adverse events that have been reported are urinary tract infections, which frequently occur in patients with autonomic synucleinopathies owing to bladder dysfunction [18].

It has been recognized that symptomatic OH is associated with poor quality of life and higher mortality rates in patients with PD, which has led to more research in this area. Newer drugs like ampreloxetine are being trialed, but no extensive literature comparing it with existing drugs could be found. Consequently, we conducted a detailed literature review of randomized controlled trials in order to explore and compare the efficacy and safety of droxidopa and ampreloxetine, novel drugs to treat symptomatic nOH in patients with autonomic synucleinopathies. We aim to compare a newer drug (ampreloxetine) with an existing standard of care (droxidopa).

## Review

Methods

Search Strategy

We conducted a literature review to find relevant articles by searching through the PubMed database. The search was conducted from mid-February to mid-March 2023 by two authors. All conflicts were resolved via discussion with a senior author. The terms "Parkinson's disease," "Parkinsonism," "droxidopa and ampreloxetine" and "orthostatic hypotension treatment" were combined in a search for original or review articles. Then, the above keywords were used to find articles and search references to find relevant research articles. We used the snowballing technique to include articles. For this narrative review paper, we only used peer-reviewed articles that have already been published or are about to be published. After looking at review articles, original research, and case reports, the current knowledge on this topic was divided into several sections, including "definition," "symptoms," "epidemiology," "pathophysiology," "neurological orthostatic hypotension," "non-neurological orthostatic hypotension," "prodromal OH," and "non-pharmaceutical and pharmaceutical treatments."

Data Screening and Eligibility

We included articles that fulfilled the following inclusion criteria: (1) published PubMed-indexed randomized controlled trials (open-labeled and double-blinded), (2) studies that included human data and not animal model studies, (3) studies that included adult patients, (4) studies with patients who were diagnosed with nOH, (5) studies involving droxidopa or ampreloxetine as a treatment modality for OH, and (6) studies in English language only.

Articles with the following characteristics were excluded: (1) invitro studies; (2) case reports, case series, observational studies, triple-blinded studies, systematic reviews, and meta-analysis; (3) studies on pediatric, geriatric, and/or obstetric patients; (4) studies using other drugs as treatment modalities for OH; (5) studies that are not in English; and (6) studies that were not peer-reviewed.

In doing so, we had a total of 10 articles with eight being on droxidopa [19-26] and two on ampreloxetine [18,27]. These were the final articles that were included in the quantitative analysis as shown in Table 1.

**Table 1:** List of all studies that were referred

Title	DOI	First Author
Droxidopa for the short-term treatment of symptomatic neurogenic orthostatic hypotension in Parkinson’s disease (nOH306B) [21]	10.1002/mds.26086	Robert A Hauser
Integrated analysis of droxidopa trials for neurogenic orthostatic hypotension [23]	10.1186/s12883-017-0867-5	Italo Biaggioni
Droxidopa for neurogenic orthostatic hypotension: a randomized, placebo-controlled, phase 3 trial [25]	10.1212/WNL.0000000000000615	Horacio Kaufmann
Droxidopa in patients with neurogenic orthostatic hypotension associated with Parkinson’s disease (NOH306A) [20]	10.3233/JPD-130259	Robert A Hauser
Randomized withdrawal study of patients with symptomatic neurogenic orthostatic hypotension responsive to droxidopa [26]	10.1161/HYPERTENSIONAHA.114.04035	Italo Biaggioni
Effects of the novel norepinephrine prodrug, droxidopa, on ambulatory blood pressure in patients with neurogenic orthostatic hypotension [22]	10.1016/j.jash.2016.07.009	Horacio Kaufmann
Droxidopa and reduced falls in a trial of Parkinson's disease patients with neurogenic orthostatic hypotension [24]	10.1097/WNF.0000000000000168	Robert A Hauser
Safety and durability of effect with long-term, open-label droxidopa treatment in patients with symptomatic neurogenic orthostatic hypotension (NOH303) [19]	10.3233/JPD-160860	Stuart Isaacson
Pharmacokinetics and pharmacodynamics of ampreloxetine, a novel, serotonin-norepinephrine reuptake inhibitor, in symptomatic neurogenic orthostatic hypotension [27]	10.007/s10286-021-00800-x	Arthur Lo
Safety and efficacy of ampreloxetine in symptomatic neurogenic orthostatic hypotension: a phase 2 trial [18]	10.007/s10286-021-00827-0	Horacio Kaufmann

Data Collection and Analysis

Once the articles were finalized, we extracted data from all the studies. The studies were all compiled in one place and sorted according to the first author and DOI. Data were collected in the following categories when available: demographic features such as age, sex, and location; drug and placebo, if applicable with dosages; ​​​​​duration of the study; results in comparison to placebo; and adverse events.

Next, we classified the studies on the basis of the drug they were based on. We found two studies on ampreloxetine [18,27] and eight on droxidopa [19-26] (Table 1). These two drugs were later compared and analyzed on the basis of the collected respective study results. We tabulated the data using Microsoft Excel. Referencing was done according to guidelines using EndNote.

This study did not require ethical approval as data were obtained from already available databases, and patients were not directly involved.

Results

Demographic Analysis

Based on our eligibility criteria, we included a total of 10 studies with eight studies focused on droxidopa [19-26] and two on ampreloxetine [18,27]. Our review included a total of 1347 participants from all the included studies. Many studies did not account for the demographic data of the participants who dropped out of the studies. Out of the total, 1326 (98%) completed the study; 804 (61%) participants were males, and 522 (39%) were females. All participants were above 18 years of age, with an age range of 41-92 years for studies [18,23-27] that reported specific data on age. Eight of the 10 studies were conducted over a period of four to 25 weeks [18,20-24,26,27], while two studies were conducted over a relatively longer duration of one to two years [19,25]. Demographic analysis is summarized in Table 2.

**Table 2:** Demographics

Number of Participants	Age of Participants	Sex With Numbers	
		Males	Females
1347 total and 1326 completed the study	All participants were above 18 years of age, with an age range of 41-92 years for studies	804	520

Ampreloxetine

We found a total of two published clinical trials on ampreloxetine (Table 3).

**Table 3:** Ampreloxetine analysis NE: Norepinephrine.

Study Name	Dosage	Drug	Any Placebo	Results in Comparison With Placebo
Pharmacokinetics and pharmacodynamics of ampreloxetine, a novel, selective norepinephrine reuptake inhibitor, in symptomatic neurogenic orthostatic hypotension [27]	1-20 mg per day	Open-label ampreloxetine	Matched placebo during the 1st phase only	Geometric mean ratio (GMR) of plasma NE concentrations on day 29 relative to the pre-dose baseline was 1.71 (95% CI: 1.28–2.29, p < 0.005).
Safety and efficacy of ampreloxetine in symptomatic neurogenic orthostatic hypotension: a phase 2 trial [18]	Phase A: 1-20 mg dose; Phase B: median dose 15 mg; Phase C: median dose 10 mg	Ampreloxetine	No placebo was used in Phase A and C but only in Phase B	During part A of the study, a seated systolic blood pressure increase of ≥10 mmHg was greater with the 5- and 10-mg ampreloxetine doses than with the placebo. In part B of the study, the increase was by 15.7 mmHg after ampreloxetine and decreased by 14.2 mmHg after the placebo.

The purpose of the first study was to understand the pharmacokinetics and pharmacodynamics of ampreloxetine in a representative target population of patients with nOH caused by PD, multiple system atrophy (MSA), or pure autonomic failure (PAF). To understand the impact of ampreloxetine on sympathetic (adrenergic) function, plasma concentrations of norepinephrine (NE) and its main intraneuronal metabolite 3,4-dihydroxyphenylglycol (DHPG) were measured. Thirty-four subjects with nOH were enrolled in phase II clinical trial (NCT02705755), where they received escalating doses of ampreloxetine for five days. Plasma ampreloxetine concentrations increased with the dose increase, and peak concentrations were observed after six to nine hours of ampreloxetine administration. In patients treated with ampreloxetine, plasma norepinephrine significantly increased by 71% (p < 0.005). These findings are consistent with long-lasting NET inhibition (norepinephrine transporter), which increases vasoconstrictor tone, thereby supporting the administration of ampreloxetine orally only once a day in patients with nOH [27].

In the second study, 34 patients were enrolled in a 25-week-long study, which was divided into three phases. Phase A was five days long, in which 34 participants received an escalating dose of ampreloxetine (dose escalation range: 1-20 mg). Phase B was a one-day, double-blinded, randomized, placebo-controlled study of 10 participants, in which five were administered ampreloxetine with a median dose of 15 mg, and five participants were administered with a matching placebo. Seated blood pressure increased by 15.7 mmHg four hours after ampreloxetine and decreased by 14.2 mmHg after the placebo. Phase C was a 20-week, open-label, steady-state extension phase followed by a four-week withdrawal (washout). Twenty participants received ampreloxetine with a median dose of 10 mg.

Compared to placebo, ampreloxetine doses between 5 and 10 mg had the highest responders. Standing systolic blood pressure increased by 11 ± 12 mmHg. Improvements in the OHSA (Orthostatic Hypotension Symptom Assessment) and OHDAS (Orthostatic Hypotension Daily Activity Scale composite scores) were seen. Minimal adverse effects of ampreloxetine were noted throughout the treatment duration [18].

Droxidopa

Eight studies published between 2014 and 2019 (a combination of double-blind [DB] and open-label [OL] placebo-controlled studies) were included in our study. We have summarized the findings in Table 4.

**Table 4:** Droxidopa analysis SBP: Systolic blood pressure; DBP: Diastolic blood pressure; OHQ: Orthostatic Hypotension Questionnaire; OHSA: Orthostatic Hypotension Symptom Assessment; BP: Blood pressure; DB: Double-blind.

Study Name	Drug	Dosage	Any Placebo	Results in Comparison to Placebo
Safety and durability of effect with long-term, open-label droxidopa treatment in patients with symptomatic neurogenic orthostatic hypotension (NOH303) [19]	Open-label droxidopa	100–600 mg three times daily	Matched placebo during the DB phase	Droxidopa maintained baseline standing SBP, and DBP increases. SBP increase ranged from 6.9 (17.5) to 14.0 (22.5) mmHg over the mean (SD) standing SBP at a baseline of 87.9 (17.5). Standing DBP increased from 57.6 (11.2) to 2.3 (11.1) to 6.9 (12.5) mmHg over. Droxidopa had a lower mean change in the OHQ composite score than placebo (0.57 units versus 0.90 units), but this was not statistical significance.
Droxidopa in patients with neurogenic orthostatic hypotension associated with Parkinson’s disease (NOH306A) [20]	Double-blind droxidopa	100-600 mg	Matched placebo in both phases	With droxidopa, the mean improvement in OHQ score was −2.2 (2.4) versus −2.1 (2.5) in the placebo group. The difference between treatment groups was significant at week 1, at +8.4 (17.4) versus −4.1 (20.5) mmHg (p = 0.04), and among droxidopa recipients.
Droxidopa for the short-term treatment of symptomatic neurogenic orthostatic hypotension in Parkinson's disease (nOH306B) [21]	Double-blind droxidopa	100–600 mg three times daily	Matched placebo in both phases	In the droxidopa group, there were favorable changes from baseline in OHSA and s-SBP at weeks 2 through 8 of treatment, but these were not statistically significant. Also, the droxidopa group had 68% of fewer patient-reported falls than the placebo group (229 vs 716).
Effects of the novel norepinephrine prodrug, droxidopa, on ambulatory blood pressure in patients with neurogenic orthostatic hypotension [22]	Open-label droxidopa	100–600 mg three times daily	Matched placebo in week one only	Droxidopa leads to increased ambulatory BP during the day.
Integrated analysis of droxidopa trials for neurogenic orthostatic hypotension [23]	Double-blind droxidopa	429 ± 163 mg	Matched placebo during DB phase	Droxidopa significantly reduced the OHQ composite score (-2.68 ± 2.20 vs -1.82 ± 2.34 units); 68% fewer falls in the droxidopa group.
Droxidopa and reduced falls in a trial of Parkinson's disease patients with neurogenic orthostatic hypotension [24]	Double-blind droxidopa	100–600 mg 3 times daily	Matched placebo in both phases	Fall rate in the droxidopa group = 0.4 per patient-week, fall rate in the placebo group = 1.05 per patient-week.
Droxidopa for neurogenic orthostatic hypotension: a randomized, placebo-controlled, phase 3 trial [25]	Double-blind droxidopa	100-600 mg 3 times a day	Matched placebo during DB phase	There was an improvement in the mean OHQ composite score with droxidopa over placebo. With droxidopa, the mean standing systolic BP increased by 11.2 vs 3.9 mm Hg (p < 0.001).
Randomized withdrawal study of patients with symptomatic neurogenic orthostatic hypotension responsive to droxidopa [26]	Double-blind droxidopa	389.6 ± 180.9 mg range, 100–600 mg 3× daily	Matched placebo during DB phase	The symptoms worsened by 1.3 ± 2.8 units in the droxidopa group versus 1.9 ± 3.2 in the placebo group (p = 0.509). In the droxidopa group, there were mean changes of 4 out of 5 OHSA symptom ratings but not statically significant.

Efficacy measures included Orthostatic Hypotension Questionnaire (OHQ) scores, blood pressure (supine and standing), and clinical global impression-severity scale (CGI-S) and clinical global impressions-improvement scale (CGI-I), both self-reported and reported by physicians. OHQ scores were reduced by -2.2 versus -2.1 in the first study [20], favored droxidopa by 0.90 in the second study [25], and had >50% reduction from baseline at the end of the first month (-3.29 units) of treatment that persisted for the remainder of the 12-month study in a third study [19]. An integrated analysis of three clinical trials on droxidopa, which was published in 2017, yielded a reduction in OHQ composite score (-2.68 versus -1.82 units in placebo) [23]. In the same study, the dizziness/lightheadedness score was reduced by -3.0 versus -1.8 units, and improvement was seen in three of five other symptom assessments (visual disturbances, weakness, and fatigue) [23]. In another study, OHSA item 1 (dizziness/lightheadedness, the primary efficacy endpoint) increased (i.e., symptom worsened) by 1.3 ± 2.8 units in the droxidopa group versus 1.9 ± 3.2 in the placebo group. However here, the persistence of symptomatic improvement during the withdrawal phase even in the placebo group caused results to be statistically insignificant due to the reduced power of the study design [26].

The first study reported fluctuant blood pressure. The difference between treatment groups seemed significant at week 1, at +8.4 versus −4.1 mmHg in favor of droxidopa recipients. Droxidopa recipients also showed improvement in the standing systolic blood pressure (s-SBP) at weeks 1, 2, and 4, implying a hemodynamic benefit initially, but by week 8, the placebo group also exhibited improved standing systolic blood pressure [20]. In the second study, standing systolic BP increased by a mean of 11.2 mmHg in droxidopa recipients versus 3.9 mmHg in placebo recipients. Thereby, at the endpoint, the mean standing systolic BP values were 107.4 and 101.8 mmHg, respectively [25]. One study found that the mean changes from baseline in lowest s-SBP favored the droxidopa group at week 2 by 6.1 mmHg and at week 8 by 4.1 mmHg [21]. A consolidated analysis of three clinical trials also reported that droxidopa increased upright SBP by 11.5 versus 4.8 mmHg for the placebo. Rates of supine hypertension were also slightly higher in patients receiving droxidopa (≤7.9% vs ≤4.6% for placebo) [26]. An ambulatory BP study (off-drug) published in 2016 stated that droxidopa led to increasing ambulatory BP during the day (systolic BP increased by 7.3 mm Hg and mean diastolic BP increased by 4.8 mm Hg) with a low risk of supine hypertension at night. Ambulatory BP monitoring, however, may help to identify the minority of patients who do develop nocturnal supine hypertension in larger sample sizes [22].

Another study used fall analysis as a measure of the efficacy of the drug. Droxidopa yielded a fall rate of 0.4 falls per patient-week versus 1.05 falls per patient-week in the placebo group, concluding in a relative risk reduction of 77%. Fifty-four patients on droxidopa reported 245 days with at least one fall. Within those days, the worst fall of the day (which could have been the only fall of the day) was linked to lightheadedness in 46.5%, loss of consciousness in 9.4%, and FOG (freezing of gait) in 26.9% of the cases. Meanwhile, in the placebo group, 63 patients reported 372 days with at least one fall-related injury that was less prevalent in the droxidopa group at 16.7% versus 26.9% in the placebo group. The study surmised that the observed reduction in falls in the droxidopa group is due to the improvement in OH, but other mechanisms such as improvement in freezing or attention might also play a role [24]. In a second study, the droxidopa group had 229 reported falls, whereas the placebo group had 716 patient-reported falls in the same study [21]. About 68% of fewer falls were reported in patients treated with droxidopa in contrast to placebo in the integrated analysis of clinical trials [23].

Adverse Effects

Based on our review, the most frequently reported adverse events in the droxidopa studies were headaches (9.68%), followed by dizziness (6.48%), nausea (4.17%), and urinary tract infections (3.22%) [19-26]. In ampreloxetine, the most commonly documented adverse events were urinary tract infections (23.8%), which often affect patients with autonomic synucleinopathies due to bladder dysfunction, particularly those with MSA, who constituted the majority of the study participants. This was then followed by hypertension (19%) and headache (14.3%) [18].

Discussion

Summary of Findings

The authors reviewed 10 studies that are clinical trials, eight of which focused on droxidopa and two others focused on ampreloxetine. This has been illustrated in Table 1. There were a total of 1326 participants who completed the study (804 males and 522 females, all above 18 years old) as seen in Table 2. Most studies had a duration of four to 25 weeks, with two longer studies conducted over one to two years. Ampreloxetine's pharmacokinetics and pharmacodynamics were evaluated in a representative patient population with nOH, where it was observed that the plasma norepinephrine level increased, while the intraneuronal metabolite 3,4-dihydroxyphenylglycol decreased after ampreloxetine. In addition, ampreloxetine safety and its impact on blood pressure were assessed. Adverse effects of droxidopa included headaches, dizziness, nausea, and urinary tract infections, while the most commonly reported adverse effects of ampreloxetine were urinary tract infections.

Pathophysiology

The pathophysiology of nOH in PD is due to a combination of baroreceptor failure and cardiac sympathetic denervation [28]. The baroreceptor reflex is important in the short-term regulation of blood pressure. It has afferent, central, and efferent limbs [29].

Normally, upon standing, there is a pooling of about 500-800 mL of venous blood in the lower extremities, which reduces the venous return and the stroke volume of the heart causing a drop in BP. This is sensed by baroreceptors in the carotid sinus and aortic arch and relayed to the nucleus tractus solitarius (NTS). Projections from NTS to the rostral ventrolateral medulla (RVLM) and subsequently to the postganglionic neurons in the sympathetic chain via the intermediolateral cell column of the spinal cord modulate the peripheral vasoconstriction and increase in BP [30,31]. Sympathetic neurons from RVLM to the heart increase heart rate and myocardial contractility [31].

The loss of postganglionic sympathetic neurons and cardiac sympathetic denervation secondary to intracellular accumulation of alpha-synuclein [32] leads to a blunted baroreceptor response and, as a consequence, nOH. This has been confirmed by multiple studies using cardiac neuro-imaging, which has shown decreased uptake of sympathetic agents like 123I metaiodobenzylguanidine (MIBG) [33]. Furthermore, norepinephrine levels double when a person stands from a supine position; this is not seen in PD due to a loss of postganglionic sympathetic innervation [31].

Treatment options for patients with nOH

Non-pharmacological Treatment Options

Initial measures to treat the symptoms of nOH generally encompass reviewing medications and withdrawing the drugs that exacerbate nOH and other non-pharmacological interventions as discussed below [34]. The non-pharmacological approach should be applied before trying medications to control nOH symptoms; these involve increasing fluid intake and salt, physical counter-pressure maneuvers, compression garments, sleeping in head-up positions, and lower-body strength training [35,36].

The most effective strategy is to limit the drop in blood pressure using bolus fluid intake (relatively 500 mL in five minutes) and abdominal compression. However, fluid ingestion should be used with caution in patients with heart failure and renal insufficiency [37]. Physical maneuvers such as toe-raising, crossing legs, and squatting can increase venous return to the heart, thus elevating cardiac output and improving nOH symptoms [38]. High temperature accelerates vasodilation; patients may get the advantage of limiting their body exposure to hot weather and excessively hot water contact [39]. Although a vast majority of patients do not benefit from non-pharmacological therapies, their implementation is essential [40].

Pharmacological Treatment Options

When medication adjustments and nonpharmacologic management fail to alleviate nOH symptoms, pharmacologic therapy is recommended to augment BP [4,15-17]. One strategy is to increase the peripheral vascular resistance with sympathomimetic agents (midodrine or droxidopa), expand the red cell mass, or increase the intravascular volume with the synthetic mineralocorticoid fludrocortisone. All available drugs that raise BP in the standing position also raise BP in the supine position, thereby increasing the risk of worsening SH [4].

Sympathomimetic Agents

Sympathomimetic agents, including midodrine and droxidopa, are the current standard of care in patients with disabling nOH. However, patients with pre-existing cardiac or renal diseases should not be put on treatment with these agents [17].

Midodrine

Midodrine is a peripheral alpha-1 agonist. Midrodine increases vascular resistance, thereby improving the standing systolic BP in patients with nOH. It has a dose-dependent response. Some adverse events noted are an increased risk of supine hypertension, piloerection, scalp tingling, and urinary retention. The typical dosing of midodrine is 2.5-15 mg three times in a day (preferably during the awake periods) [4,15].

Norepinephrine Reuptake Inhibitors

Atomoxetine and ampreloxetine are norepinephrine membrane transport inhibitors. They increase norepinephrine in the neurovascular junction [4]. In patients with autonomic impairment of the central system, norepinephrine reuptake inhibitors induce only peripheral vasoconstriction, making them ideal for patients with MSA.

Atomoxetine

Atomoxetine is a short-acting norepinephrine reuptake inhibitor that, when given 10-18 mg twice daily, increases standing blood pressure and relieves the nOH symptoms [4].

Off-label medications

Fludrocortisone

Fludrocortisone is a synthetic mineralocorticoid that induces sodium and water retention, thereby increasing plasma volume [4,17]. The typical dose is 0.05-0.2 mg daily [15]. Fludrocortisone may cause supine hypertension, hypokalemia, headache, ankle edema, myocardial fibrosis, and hypokalemia [4,15]; electrolyte monitoring is recommended when using this drug [17].

Pyridostigmine

Pyridostigmine is an inhibitor of cholinesterase that potentiates cholinergic neurotransmission in the autonomic ganglia, both sympathetic and parasympathetic [4,15]. Studies have shown that pyridostigmine increases, on average, only 4 mmHg in SBP.

Other Medications

Other agents such as the vasopressin analog desmopressin (DDAVP), the centrally acting α2-antagonist yohimbine, the ergot alkaloid dihydroergotamine, the non-selective adrenergic agonist pseudoephedrine, the serotonin uptake inhibitor fluoxetine, and indomethacin are superseded and rarely prescribed today due to their problematic adverse effects [4]. Such pharmacological options can be considered in selected cases with refractory OH if other agents failed or caused side effects [17].

Droxidopa

Droxidopa is a prodrug of norepinephrine [41]. Administration of droxidopa, a synthetic amino acid that is decarboxylated to norepinephrine by the enzyme L-aromatic amino acid decarboxylase, increases the standing blood pressure, ameliorates the symptoms of OH, and improves the ability to change posture in patients with nOH due to degenerative autonomic disorders like Parkinson’s disease [42-44]. Droxidopa only acts peripherally and does not cause the blood-brain barrier [41-43,45].

Droxidopa was approved for use in the United States in 2014. Oral bioavailability is 90% with a biological half-life of two to three hours. The metabolism of droxidopa is mediated by the catecholamine pathway and not by the cytochrome P450 system. Droxidopa is mainly excreted in the urine, with the main metabolite being 3-O-methyl-dihydroxy-phenylephrine [42,44,46]. No dose adjustment is required for mild to moderate renal impairment (GFR > 30 mL/min) [44,45]. Since its approval, there have been no published reports of droxidopa hepatotoxicity [41,46].

Common side effects of this drug include headache, dizziness, nausea, and rarely neuroleptic malignant syndrome [41,42]. Co-administration with medications that increase sympathetic (adrenergic) effects, including increased blood pressure and heart rate, may increase the risk for supine hypertension. Because of this, supine blood pressure should be monitored before and during treatment and more frequently when doses are increased. If supine hypertension cannot be managed by elevation of the head of the bed, reducing the dosage or finally discontinuing droxidopa would be the next step [42-44]. Due to its sympathomimetic effect, droxidopa may also exacerbate the existing ischemic heart disease, arrhythmias, and congestive heart failure [42,43].

There is no available data on the use of droxidopa in pregnant women and the risk of major birth defects or miscarriage. There is no information on the presence of the drug or its active metabolite(s) in human milk, its effects on breastfeeding, or its effects on milk production/excretion. Due to the potential for serious adverse reactions, including reduced weight gain in breastfed infants, women are advised not to breastfeed during treatment with droxidopa [43,44].

Ampreloxetine

Ampreloxetine is a selective norepinephrine transporter (NET) inhibitor. It boosts the norepinephrine bioactivity at the neurovascular junction, thereby increasing the outcome of the residual postganglionic sympathetic neurons. This is the case, particularly on standing up when a baroreceptor reflex is triggered [47].

Ampreloxetine produces a constant state of NET inhibition with a half-life of 30-40 hours; thus, it could be useful to treat nOH when given once daily at the 10-mg dose. Ampreloxetine does not require multiple-dose monitoring, and its pharmacokinetics and pharmacodynamics have been proven to be safe in relation to renal functions [48,49]. Generally, ampreloxetine is well-tolerated; one of its most common adverse events is urinary tract infections [50].

Limitations

Although our review has been conducted on an important topic and addresses a gap in the existing literature, our study was not without limitations. One limitation is the fact that it was not a systematic review and that we did not include any statistical analysis for a meta-analysis. However, it is one of the few studies reviewing the management of OH in Parkinson's disease and discussing the newer medications, namely, droxidopa and ampreloxetine. Conducting a meta-analysis requires various tools like different software, a good library, a statistician, etc. Although we did not look at multiple databases, we did a targeted review of the topic, focusing on articles on specific drugs and comparing an existing standard treatment with a relatively newer drug form. We also tried to overcome this limitation by thoroughly screening through literature to only include peer-reviewed studies. Second, because our review includes all studies that were randomized clinical trials, we were not able to include patient-reported outcomes. Future studies should gather qualitative data gained from patient insights. Another limitation of our study is that it included only adult participants. However, there is not a lot of existing data on children or pregnant patients because of the nature of the disease.

## Conclusions

This targeted literature review demonstrates the efficacy of droxidopa and ampreloxetine in managing nOH and improving the quality of life in patients with Parkinson's disease or other neurological diseases with nOH. Of the two drugs discussed, droxidopa showed prudent effects in managing postural hypotension. At the same time, ampreloxetine led to persistently elevated norepinephrine levels in the plasma, thus improving OH. However, further studies need to be conducted to evaluate the pharmacological profile of ampreloxetine with respect to the management of nOH. Similarly, rigorous studies involving larger sample sizes, larger scale clinical trials, a meta-analysis comparing the new drug with a standard of care, and patient-reported outcomes are warranted to have more definitive findings.
